# Gout and AA-Amyloidosis: A Case-Based Review

**DOI:** 10.31138/mjr.32.1.74

**Published:** 2021-02-15

**Authors:** Margarita Aleksandrovna Gromova, Vladimir Viktorovich Tsurko

**Affiliations:** 1 Pirogov Russian National Research Medical University (RNRMU), Moscow, Russian Federation; 2 I.M. Sechenov First Moscow State Medical University (Sechenov University), Moscow, Russian Federation

**Keywords:** Gout, amyloid A, amyloidosis, treatment, colchicine, allopurinol, canakinumab

## Abstract

**Background:**

AA-amyloidosis complicates many chronic infections and inflammatory diseases, including rheumatoid arthritis, ankylosing spondylitis, and psoriatic arthritis, but its relationship to gout is extremely rare. As it is unknown definitely what the pathophysiological connections between gout and amyloidosis are, treatment issues of the diseases are open for discussion.

**Aim:**

To establish a link between gout and AA-amyloidosis, and to improve the quality of treatment in patients suffering from gout and AA-amyloidosis.

**Methods:**

We reviewed the English-language literature sources, searching not only for rare cases of the combination of gout and AA amyloidosis, but also detailed descriptions of the medical treatments for the two pathologies.

**Results:**

By July 2020, we had identified 14 cases describing AA amyloidosis in patients with gout. Most of those patients had been suffering tophaceous gout for at least 10 years, and were prescribed various methods of treatment; however, not all patients took colchicine regularly. In some cases, therapy with allopurinol and colchicine was effective against attacks of gouty arthritis, although amyloidogenic inflammation was not controlled sufficiently. However, there were no cases that described in detail the successful treatment of both diseases. Besides those 14 patients described in literature, we examined one more patient with amyloidosis that is secondary to gout, in whom the protein of amyloid A (AA) had affected the kidneys, intestines, and adrenal glands. The patient has been successfully treated with the combination of canakinumab, prednisone, colchicine and allopurinol.

**Conclusion:**

Clinicians should be aware that patients may have atypical combinations of diseases like gout and amyloidosis. The obtained results help to explain some pathogenic processes associated with AA-amyloidosis. Further research is necessary to confirm the effectiveness of different treatment options such as lifestyle biologic agents or other medicines with anti-inflammatory properties.

## INTRODUCTION

AA-amyloidosis, previously known as secondary or reactive amyloidosis, is a long-recognized severe complication of some chronic inflammatory diseases.^1^ Organ damage in AA-amyloidosis results from the extracellular deposition of the soluble acute-phase reactant serum amyloid A (SAA) protein as insoluble amyloid fibrils. A sustained high concentration of SAA produced by the liver during a chronic inflammatory state is the prerequisite for developing AA-amyloidosis.^2^ Patients are at risk of developing AA-amyloidosis if they suffer from such chronic inflammatory diseases as seropositive and seronegative chronic polyarthritis, rheumatoid arthritis, ankylosing spondylitis, juvenile chronic arthritis, psoriatic arthropathy, Reiter’s syndrome, and also from chronic inflammatory bowel diseases such as Crohn’s disease, chronic ulcerative colitis, from auto inflammatory diseases such as gout in severe recurrent tophus form, from family periodic fever, ie, periodic disease cryopyrinopathy, from periodic syndrome receptor associated factor, from tumour necrosis, hyperimmunoglobulinaemia D, from chronic suppurative diseases such as tuberculosis, bronchiectasis disease, from osteomyelitis, etc., and from large malignant tumours.^3–5^

The risk of AA-amyloidosis increases in patients with chronic inflammatory diseases with persistent increased markers of the acute phase of inflammation (C-reactive protein, SAA), and in the presence of anaemia of chronic diseases (with increased levels of blood ferritin) especially if they are in combination with articular syndrome (synovitis). It should be noted that gout as a cause of secondary amyloidosis is extremely rare.^5,6^

The diagnosis of amyloidosis can be reliably confirmed with histological research. The detection of the characteristic phenomenon of metachromatic birefringence upon microscopic examination of preparations stained with Congo red under a polarizing microscope is considered the “gold standard” of diagnosis. The next stage of verification diagnosis (determination of the type of amyloidosis) is immunohistochemical study using antisera to the main types of amyloid protein. However, the study does not give information on the extent or progression of the disease. Nuclear imaging modalities play an important role in primary diagnosis and monitoring of illness, based on radiotracer accumulation within the sites of the disease. For example, the ^123^Iodine serum amyloid protein (SAP) is used for detection of amyloid with high sensitivity and specificity. A great value of SAP scintigraphy 24 hours after intravenous administration of ^123^I-SAP showing the presence of SAP is that the possibility to exclude systemic deposits in case of a localized disease. Although amyloidosis is always diagnosed on the basis of positive biopsy results, the advantage of nuclear imaging is its ability to noninvasively assess the extent of involvement (whole-body scintigraphy) and the lack of potential complications (bleeding or perforation, infection). The drawback is that ^123^I-SAP scintigraphy is expensive and not readily available.^7^

Currently, specific treatments have been developed for most forms of amyloidosis, although some of these treatments are still being studied. Colchicine is effective in familial Mediterranean fever with AA amyloidosis (0.6 mg orally 1–2 times/day). Treatment of other types of AA is aimed at stopping the underlying disease: infection, inflammatory disease, or cancer. To interrupt signal transmission from cytokines, the following drugs are used: Anti-IL-1-, anti-IL-6- and anti-tumour necrosis factor (TNF). They reduce the inflammatory process leading to the production of SAA in the liver, although, in the literature we have not found any description of the effect of such treatment in patients with gout complicated by reactive amyloidosis.^8–10^

Our research aim is to establish a link between gout and AA-amyloidosis and to improve the quality of treatment in patients with gout-accompanied AA-amyloidosis.

We reviewed the English-language literature sources describing not only rare cases of gout in combination with AA-amyloidosis, but also detailed descriptions of the two pathologies treatment. A literature search was conducted using the MEDLINE/PubMed and Scopus databases, that consisted only of published data and included clinical cases, original studies, and existing review articles. The following search string was used: chronic gout, amyloid A, amyloidosis, treatment, colchicine, allopurinol, canakinumab. Articles were checked for relevance, and only those related to the topic of our study were included in the review.

## CASE PRESENTATIONS

In addition to our case, by July 2020, we had found 14 cases in which reactive amyloidosis in patients with gout were described.^11–20^ The characteristics of patients reported in the English literature and the case of our patient (totally 15 patients) are shown in **Table 1**. Most of the patients had been suffering from chronic tophaceous gout for at least 10 years, and they had undergone various treatment methods, but did not use colchicine regularly. We focused on gout as the cause of AA amyloidosis and its treatment. In some cases, therapy with allopurinol and colchicine were effective against attacks of gouty arthritis, although it did not sufficiently control amyloidogenic inflammation. However, there were no descriptions detailing the successful treatment of both diseases with a single medicine or a combined therapy. We report that canakinumab, in combination with prednisone, colchicine, and allopurinol can be used as a novel adjunctive therapy to successful treatment of gout and secondary amyloidosis.

**Table 1.** Characteristics of patients.

**Patient number and reference**	**Sex**	**Age, year**	**Duration of gout, year**	**Severity of gout**	**history Family**	**Site of amyloid deposition**	**Treatment**
1^11^	Male	42	25	Chronic tophaceous gout		Generalised	Azathioprine, dialysis
2^11^	Male	23	9	Chronic tophaceous gout	+	Kidneys	Probenecid, dialysis
3^11^	Male	32	4	Monoarthritis		Kidneys	Sulphinpyrazone
4^12^	Male	72	37	Chronic tophaceous gout		Kidneys	High doses of salicylates, allopurinol, dialysis
5^13^	Male	46	32	Chronic tophaceous gout	+	Kidneys, adrenals, testicular blood vessels	Only colchicine during an attack
6^13^	Male	39	3	Oligoarthritis		Kidneys	Analgesics and probenecid
7^14^	Male	85	10	Chronic tophaceous gout		Rectum, kidneys probable	Colchicine started after diagnosis of amyloidosis
8^15^	Male	49	20	Chronic tophaceous gout		Kidneys, liver, subcutaneous fat	No use of colchicine at all
9^16^	Male	61		Chronic tophaceous gout		Kidneys	
10^17^	Male	46	20	Chronic tophaceous gout		Rectum, kidneys probable	No use of colchicine at all
11^18^	Male	56	15	Chronic tophaceous gout	+	Kidneys	
12^18^	Male	44	10	Chronic tophaceous gout	+	Rectum, kidneys	Allopurinol after diagnosis of amyloidosis Allopurinol, colchicine started
13^19^	Male	57	20	Chronic tophaceous gout		Kidneys	Only colchicine during an attack
14^20^	Female	47	1	Oligoarthritis		Kidney, liver	Allopurinol, colchicine and prednisolone | anakinra and tocilizumab, infliximab | dialysis and medical nephrectomy
15	Male	62	36	Chronic tophaceous gout		Kidneys, gastrointestinal tract; adrenal glands	Canakinumab, prednisone, colchicine, allopurinol, sotalol

### Description of the clinical case of the 15^th^ patient

A 62-year-old European man was admitted to hospital because of severe impairment of renal function. It is known from his anamnesis that for over 26 years he had been suffering from the tophus form of gout. The patient regularly took Allopurinol 300 mg/d, but he did not follow the special diet. Periodically, he was disturbed by the pain in the knee joints of the aching nature. Once every 6 months the patient was injected Betamethasone intraarticularly and he took NSAIDs independently.

In 2000, urolithiasis was detected and there was an episode of renal colic with a calculus discharge. The patient was constantly followed up by a urologist in hospital and he was treated with a conservative therapy. Beginning 2003, he began to notice an increase in blood pressure, up to 190/100 mm Hg, but he did not take any antihypertensive therapy then.

In 2014, due to the increase in proteinuria, and in creatinine level up to 137–176 μmol/l, ESR to 99 mm/h, CRP to 40 mg/l, he was recommended to refrain from taking NSAIDs. He began to take prednisone 10 mg/d with a gradual decrease in dose up to 5 mg/day. The patient has been aware of an increase in creatinine level for about 4 years, and he has been supervised by a nephrologist. There was an attempt to take nephroprotective therapy with angiotensin-converting enzyme inhibitors. However, because of the patient’s tendency for hypotension, the medicine was cancelled.

In May 2017, there was inpatient treatment in the cardiology department as atrial fibrillation had developed in the patient. He was prescribed Rivaroxaban. In June 2017, the patient was admitted to the urology department due to tamponade of the bladder. His creatinine became 230 μmol/l. Anticoagulant therapy was cancelled. The patient was sent to the therapeutic department for further examination. During the examination, the patient’s haemoglobin was 118 g/l, ESR – 66 mm/h, proteinuria was 3.0–7.98 g/l, leukocyturia was up to 750 cells/ μl, erythrocyturia was 37 cells/μl, creatinine was 189 μmol/l, glomerular filtration rate (GFR) using the CKD-EPI creatinine equation was 41 mL/min, total protein was 50 g/l, albumin was 19 g/l, uric acid was 302 μmol/l, CRP was 92 mg/l. The immunochemical tests of serum proteins and urine showed no pathological gradients and monoclonal secretion was not detected. Ultrasound and computer tomography showed the kidneys were reduced in size, while their parenchyma was thinned. Stones and two complex cysts with indirect signs of haemorrhage were detected in the upper segment of the right kidney. Computer tomography of the chest showed no lymphadenopathy.

**Figure 1.**
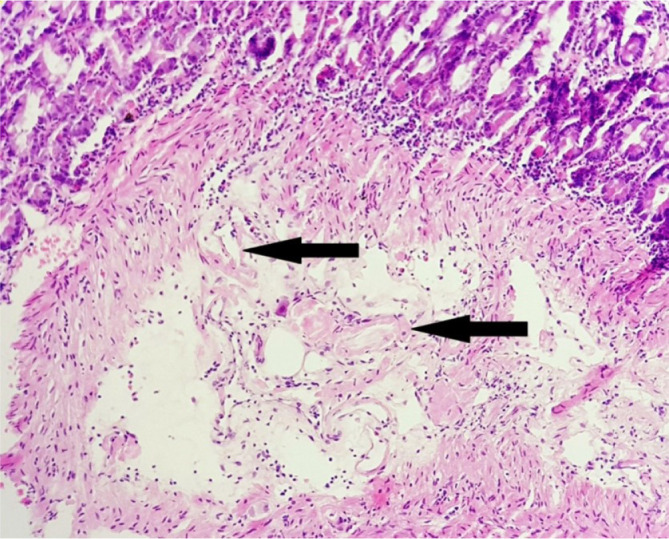
Submucosal layer. Hematoxylin-eosin (HE) staining shows eosinophilic (pink) masses in vessel walls in submucosal vessels (HE 200×).

**Figure 2.**
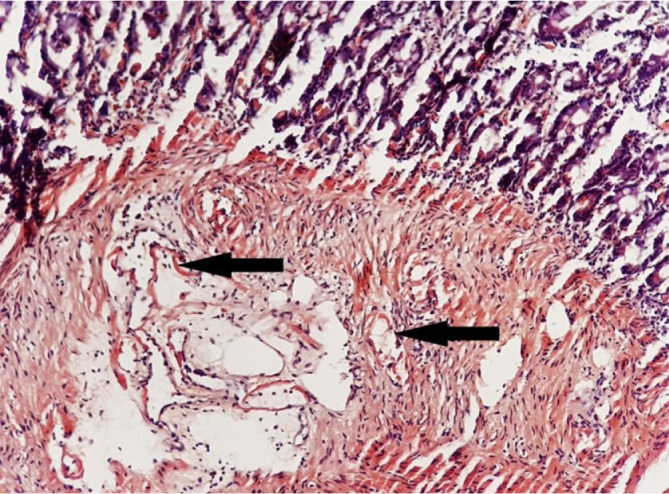
Submucosal layer. There is a deposition of homogeneous brick red masses which are painted with Congo red (CR 200×).

**Figure 3.**
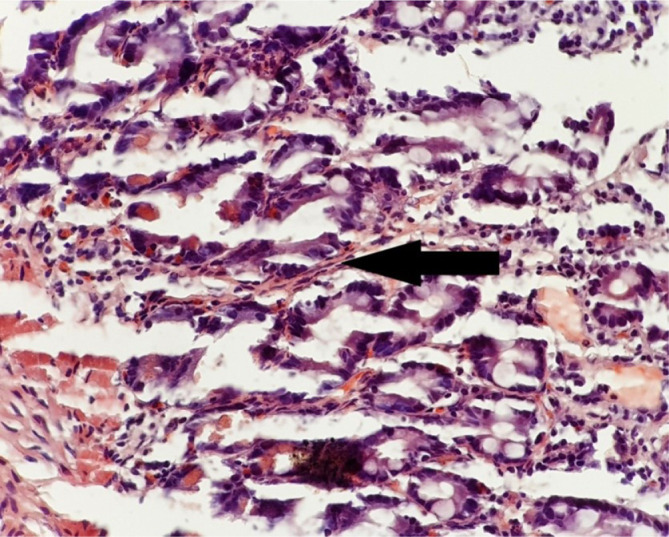
Lamina propria of the mucosal membrane. There is a deposition of homogeneous brick red masses which are painted with Congo red (CR 400×).

In view of a nephrotic syndrome, a long period of gout and hypotension, the patient was suspected to have amyloidosis. It was not advisable to perform a kidney biopsy considering the data of the additional examinations. Therefore, a biopsy of subcutaneous fatty tissue and a mucosal biopsy during the esophagogastroduodenos-copy examination with subsequent amyloid staining were performed. Histological examination by the polarized light showed extensive deposition of Congo red material, the indicative of severe amyloidosis (**Figures 1–3**). The deposition proved to be the type AA-amyloidosis on immune-peroxidase staining.

The patient was prescribed colchicine 1.0 per day, Sotalol 40mg twice a day. Blood pressure was controlled at 90–70 and 50mm Hg. Against the background of the treatment the level of inflammation indices remained high within three months. There was an attempt to increase colchicine up to 1.5 mg per day, but it failed because of the patient’s diarrhoea. Due to the sharp weight loss and changes in his tests, the patient was consulted by an oncologist. Positron emission tomography-computed tomography (PET-CT) was carried out to exclude paraneoplastic process and detect the smouldering gout centres supporting an acute inflammation phase. The oncopathology was excluded.

Treatment with allopurinol 100 mg/d and colchicine 1.0 mg/d is effective against gouty arthritis attacks, however it is not sufficient to control amyloidogenic inflammation. The patient’s creatinine was 259 μmol/l, GFR using the CKD-EPI creatinine equation was 20 mL/min, total ESR was 63 mm/h, hyperfibrinogenaemia was 8.44 g/l, proteinuria was 8,02 g/l, leukocyturia was up to 1110 cells/μl, erythrocyturia was 17 cells/μl, total protein was 53,4 g/l, albumin was 20,6 g/l, uric acid was 318 μmol/l, CRP was 40 mg/l. The tests showed the necessity of enhancement of the therapy with canakinumab 150 mg s/c once per month. Against the background of combined therapy, there were no negative dynamics: the patient’s creatinine was 208 μmol/l, GFR using the CKDEPI creatinine equation was 35,4 mL/min, total ESR was 35 mm/h, proteinuria was 1,9 g/l, leukocyturia was up to 30 cells/μl, erythrocyturia was 1 cells/μl, total protein was 52,5 g/l, albumin was 21,7 g/l, uric acid was 304 μmol/l, CRP was 2 mg/l.

The final diagnosis is formulated: gout, tophaceous form; chronic gouty arthritis, relapsing synovitis of the knee joints, joint function failure II. Chronic kidney disease stage IV; kidney cysts, urolithiasis, secondary arterial hypertension; secondary (AA) amyloidosis with kidney damage and the development of nephrotic syndrome, damage to the gastrointestinal tract and adrenal glands; mild anemia; persistent atrial fibrillation, chronic heart failure. Since 2018, the patient has been injected 150 mg canakinumab subcutaneously once a month. The patient constantly takes prednisone 5 mg/d, colchicine 1,0 mg/d, allopurinol 100 mg/d, sotalol 40 mg twice a day. Now his state is stabilised: renal function improved, proteinuria decreased, amyloid load regressed, inflammation and uric acid levels are normal, but, unfortunately, there was no full resolution of tophi.

### Discussion of similar published cases

Gout is characterised by a chronic intense inflammation of the joints. Amyloidosis due to gout disease is extremely rarely mentioned among the doctors, and the discussion is required. Gouty arthritis does not appear to be associated with an increased incidence of amyloidosis, as a small number of patients with amyloidosis have been reported up to now. The exact causes that associate gout and amyloidosis are unknown. However, several pathophysiological concepts have been suggested in the literature. In fact, amyloidosis as a consequence of tuberculosis^11^ developed in the patients #2 and #3 in **Table 1**. In patient #6, the appearance of amyloidosis preceded gout^13^ and was manifested both by a high sedimentation rate and massive proteinuria. Three patients, #1, #4, and #5, suffered from long-term recurrent gout with secondary infections of the tophi, that was well documented in patient #5. The destructions of tophi are believed to have caused the secondary amyloidosis. Uraemia was noted as one of the causes of the patient’s death.^11–13^ Patients #7–13 had been suffering from recurrent attacks of gout over the past 10–20 years and denied any previous illness, including tuberculosis, osteomyelitis, or any other chronic infectious or inflammatory disease. The association of gout and amyloidosis in the patients has been suggested to reflect a rare combination of frequent polyarticular gout attacks that cause a prolonged increase of SAA and Potential SAA Transformation Propensity into the conformation of the amyloid beta-folded sheet fibrils. It was noteworthy that, after colchicine administration, the patients’ attacks (#7 and #13) of polyarticular gout ceased, and an improvement in renal function and a restoration of serum albumin were observed to normal values. Conceivably, the infrequent association of gout and amyloidosis may be related, in part, to the time-honoured use of colchicine for preventing acute attacks of gout.^14–19^ Patient #14 appeared to have AA amyloidosis characterised by a nephrotic syndrome, a massive hepatosplenomegaly possibly related to hypothyroidism. The inflammatory underlying processes are poorly understood. The unspecified non-erosive oligoarthritis of recent origin first was thought to be gout, not severe, and recent enough to be considered the cause of this far-advanced AA amyloidosis. No other infectious or inflammatory causes were found except for the fact that the patient had been morbidly obese for many years, which can cause a chronic inflammatory state described above. There was no effect of the treatment; after the start of the haemodialysis and nephrectomy, the condition stabilized.^20^

Thus, current basis on the pathogenesis of reactive amyloidosis suggests several mechanisms. The amyloidogenic stimulus, chronic infection, or inflammation in man and endotoxin or casein administration in experimental animals, is believed to cause a persistent elevation of the serum precursor of amyloid, SAA, as well as an alteration of the degradation process of the precursor, presumably mediated by macrophages.^21,22^

In obesity, the systemic low-grade inflammation is caused by upregulation of macrophage activity resulting in secretion of the pro-inflammatory cytokines (IL-1, TNF- α, IL-6) and chemokines (eg, monocyte chemotactic protein-1 [MCP-1]) by adipocytes. IL-6 subsequently stimulates the hepatic production of CRP and SAA. Interestingly, this inflammatory state seems to be mainly driven by adipocytes from visceral white adipose tissue, and much less by adipocytes from subcutaneous white adipose tissue.^20^

Furthermore, it must be emphasised that acute inflammation is the hallmark of the acute tissue reaction to gout crystals. The mechanisms leading to joint inflammation in the disease first involve crystal formation and subsequent coating with serum proteins. Crystals can then interact with plasma cell membrane, either directly or via membrane receptors, leading to NLRP3 activation, proteolytic cleavage, and maturation of pro-IL1β and secretion of mature IL1β. Once released, this cytokine orchestrates a series of events leading to endothelial cell activation and neutrophil recruitment. Ultimately, gout resolution involves several mechanisms including monocyte differentiation into macrophage, clearance of apoptotic neutrophils by macrophages, production of Transforming Growth Factor (TGF-β) and modification of protein coating on the crystal surface.^23–29^

Another argument that may explain the low incidence of amyloidosis in gout is the use of colchicine.^13–15,17^ This substance has been shown to prevent casein-induced amyloidosis in mice.^30,31^ In addition, several patients with familial Mediterranean fever complicated by amyloidosis experienced a regression of albuminuria during prolonged treatment with colchicine. After all, since the majority of patients with gout are treated with colchicine only during attacks and a large number of patients take no colchicine at all, the low incidence of amyloidosis in the disease is unlikely to be associated with the drug.^32–34^ Currently there are studies confirming IL-1 inhibitors are effective for controlling attacks and inflammatory activity in patients with gout.^35–37^ Single canakinumab, a fully human anti-interleukin 1β monoclonal antibody, either doses ≥50mg or more or four 4-weekly doses provided superior prophylaxis against flares in comparison with daily colchicine 0.5mg.^38,39^ Moreover, IL-1 inhibitors reduce or stabilise amount of proteinuria and preserve renal function in short-term follow-up.^40–42^ However, we are not aware of the available data of the patients under treatment with anti-IL-6 or other biological agents in gout-induced AA amyloidosis.

Thus, we have presented our clinical observation as a rare example of systemic AA-amyloidosis development as a consequence of prolonged gout with the formation of tophi because of improper treatment. From our point of view, the main pathogenetic mechanism is associated with a chronic inflammatory process. In this case, it is a long-term recurrent gout without specific treatment, which increases the synthesis of acute-phase markers, ie a part of amyloid with AA-amyloidosis. We have described in detail the successful treatment of gout and secondary amyloidosis with canakinumab in combination with other medicines. Since there are no objective parameters of response to treatment of amyloidosis (SAP levels, scintigraphy), we report that it is a hypothesis that canakinumab might be effective for controlling secondary AA-amyloidosis, mainly based on the improvement of renal function, that is taken in combination with low doses of prednisone, colchicine and allopurinol. We should not, however, be so very enthusiastic about canakinumab, as it is very expensive and should be used only in special cases.

## CONCLUSION

Gouty arthritis, unlike other forms of chronic arthritis, is slightly associated with amyloidosis. The reasons for this exception have not been established, and further investigation is needed. The described case is extremely rare and most cases in the literature were reported decades ago. The obtained results help to explain some pathogenic processes associated with secondary amyloidosis. Clinicians should be aware that patients may have atypical combinations of diseases like gout and amyloidosis. Nowadays, gout is treated more efficiently, and cases of tophaceous gout are relatively uncommon. Our case raises awareness of such a rare association with amyloidosis and emphasizes the importance of considering the diagnosis of secondary amyloidosis in patients with gout. The treatment with biologic agents in patients with rapid deterioration of organs functions, eg, anti-IL-6, anti-IL-1 or TNF-α blockade, might still be attempted in order to decrease SAA levels in view of the underlying pathophysiology. Further research is needed to confirm the effectiveness of different treatment options such as lifestyle interventions, biologic agents or other drugs with anti-inflammatory properties.

## ABBREVIATIONS

AA: Amyloid A
CRP: C-reactive protein
GFR: Glomerular filtration rate
ESR: Erythrocyte sedimentation rate
IL: Interleukin
^123^I-SAP: ^123^Iodine serum amyloid protein
MCP-1: Monocyte chemotactic protein-1
NLRP3: NLR family pyrin domain containing 3
NSAIDs: Nonsteroidal anti-inflammatory drugs
PET-CT: Positron emission tomography-computed tomography
SAA: Serum amyloid A
TGF-β: Transforming growth factor beta
TNF: Tumour necrosis factor
